# Editorial: The Role of Chemoattractants in the Tumor Microenvironment

**DOI:** 10.3389/fimmu.2019.02671

**Published:** 2019-11-12

**Authors:** Giovanni Bernardini, Brian A. Zabel

**Affiliations:** ^1^Department of Molecular Medicine, Sapienza University of Rome, Laboratory Affiliated to Institute Pasteur Italia-Fondazione Cenci Bolognetti, Rome, Italy; ^2^Palo Alto Veterans Institute for Research, VA Palo Alto Health Care Systems, Palo Alto, CA, United States

**Keywords:** chemokines, cancer therapy, cancer metastases, anti-tumor immune response, cancer inflammation, immune suppression, chemokine receptor antagonists

Chemokines and other chemoattractants induce directional migration and activation of leukocytes and of non-hematopoietic cells by stimulating specific G protein-coupled Receptors (GPCR). Since the term “chemokine” was officially accepted as the standard nomenclature for “chemotactic cytokines” in 1992, there have been over 100,000 manuscripts published and indexed in Pubmed, ~20% of which also include the search term cancer. In this *Frontiers in Immunology* Research Topic, leading international investigators in the field have contributed seven reviews, three minireviews, and four original research articles to this Research Topic to provide a comprehensive and timely examination of the role of chemoattractants in the tumor microenvironment (TME). The collection provides an updated overview of the most relevant issues related to the complicated interaction between chemoattractants and other mediators produced by host or tumor cells that contribute to tumor development, growth, metastasis, and immune escape.

Immune cells are fundamental in shaping the balance between a tumor-promoting or tumor-suppressive microenvironment. A fundamental role is played by macrophages, usually referred to as tumor-associated macrophages (TAMs). TAMs can stimulate proliferation of tumor cells, promote angiogenesis and fibrosis and suppress the anti-tumor immune response. In addition, the combined action of TAM at the primary tumors and the so-called metastasis-associated macrophages (MAM) in the metastatic sites promote the metastatic cascade.

The contribution by Argyle and Kitamura in this collection underscore the role of chemoattractants and of their receptors in TAM and MAM accumulation in primary and secondary tumor sites, highlighting the potential therapeutic role of targeting macrophage-recruiting chemokines to prevent malignant tumor development. One key determinant of monocyte recruitment and TAM accumulation provided by the CCL2/CCR2 axis. Indeed, different tumor types can produce CCL2, even though expression of this chemokine is regulated by different means. Consistent with these observations, loss of *Ccr2* or CCL2 blockade inhibits TAM accumulation and is the most promising strategy for inhibition of immune suppression exerted by chemokines.

Ruytinix et al. discuss interesting observations indicating that macrophages recruited into tissues can be polarized toward cells able to produce pro-angiogenic and pro-fibrotic factors as well as to attract other immunosuppressive immune cells according to environmental factors, thus favoring tumor growth in primary site or seeding in distant organs. In addition to several growth factors, an important contribution on monocyte differentiation toward a pro-tumor phenotype is provided by chemokines, including CCL2 and CXCL12.

An original article from Lepore et al. describes a pro-tumorigenic role for CXCL16/CXCR6 signaling in glioma progression pointing to a critical role in immune-suppression. In a GL261 syngeneic orthotopic implantation model, CXCR6-deficient mice survived significantly longer than WT counterparts, with significantly reduced tumor volumes. Using anti-CXCL16 neutralizing antibodies, the authors discovered that glioma-secreted-CXCL16 induced an immune-suppressive gene expression signature in primary microglial cells.

Strategies able to inhibit macrophage recruiting or polarizing chemokines also create a permissive environment for immunotherapy, favoring activation of effector cells with anti-tumor activities. Nevertheless, activated CD8+ and NK cell populations rely on several receptors for their recruitment and infiltration and immunotherapeutic approaches are less effective in chemokine receptor deficient mice. Indeed, the distribution and phenotype of different NK cell subsets can be affected by specific types of tumor and its location and this often correlate to altered migration and homing. These and other aspects regulating trafficking and tissue localization of NK cells are discussed in this collection by Castriconi et al. Furthermore, by reporting evidence from the literature, Susek et al. revised the effect of CXCR1/2 and CXCR3, highlighting the importance of the formers in suppressive cell recruitment and of the latter in the generation of an effective T and natural killer cell anti-tumor response.

Since the first mechanistic study defining a protective role for leukocyte attractant chemerin in recruiting anti-tumor NK cells to melanoma lesions in 2012, there have been nearly 100 publications exploring the role of chemerin in cancer. The review by Shin et al. provides a comprehensive examination of chemerin in cancer, with a focus on mechanistic preclinical studies and functional consequences of chemerin in tumors. An original research article by Pachynski et al. indicates that chemerin gene expression is significantly downregulated in human breast cancer, which the authors hypothesize to be part of an adaptive tumor evasion strategy. Chemerin overexpression by mouse EMT6 breast cancer cells suppressed tumor growth *in vivo*, which was associated with increased CD4+ and NK cell infiltration into the tumor and mechanistically dependent on NK cells.

Many malignant tumors of non-hematopoietic origin express multiple chemoattractant GPCRs that increase the invasiveness and metastasis of tumor cells. In addition, chemoattractants also enable the interaction of tumor cells with host cells, thus promoting tumor growth and development of distant metastasis.

The review by Jacquelot et al. provides an in-depth look at the chemokines and chemokine receptors involved in melanoma progression. The expression of chemokine receptors by melanoma cells can be a determining factor in metastasis and survival outcomes, with CCR7, CCR10, and CXCR4 being particularly deleterious. The expression of certain chemokine receptors on blood or tumor infiltrating leukocyte subsets from melanoma patients or from preclinical studies can also be a determining factor in prognosis. The authors provide an up-to-date assessment of translational chemokine receptor targeting approaches in melanoma, noting the “double-edged sword” nature of this approach, in that targeting receptors expressed by melanoma may impair effective anti-tumor leukocyte functions.

Triple-negative breast cancer (TNBC) is a subgroup of diagnosed breast cancer patients without targeted therapeutic options. Notch receptor expression and activation strongly correlate with the aggressive clinicopathological and biological phenotypes of breast cancer. Two articles by Liubomirski et al.; Liubomirski et al., together point at the pro-inflammatory microenvironment, and at the Notch pathway, as targets for potential future treatments in TNBC. The authors found that TNBC from patient samples exhibited increased levels of Notch1 and Notch 3 and decreased Notch4 compared to luminal A breast cancers. Moreover, Notch1 expression correlated with TNF-alpha and CXCL8 expression. Notch 1 regulated the contact-dependent induction of CXCL8, and TNF-alpha stimulation led to activation of p65 and subsequently CXCL8 production. The authors conclude that the Notch pathway is a key mechanism for up-regulation of CXCL8 resulting in increased aggressiveness of TNBC.

In an intriguing change of pace from considering the role of chemoattractant receptors on tumor cells or leukocytes, Salazar and Zabel reviews the ways in which chemokine receptor expression by tumor endothelial cells (TEC) can support cancer progression. TEC are highly heterogeneous and express a variety of chemokine receptors such as ACKR1, ACKR3, CXCR4, CCR2, CXCR2, and CXCR3. TME-derived chemokines contribute to the morphological and phenotypic dysregulation of the vascular endothelium, leading to pro-tumorigenic angiogenesis, vasculogenesis, intussusception, vessel co-option, and/or vascular mimicry. The authors speculate that chemokine receptors may be particularly promising targets for future vascular disruption therapies based on their restricted expression (e.g., not by vital organs) and the potential for concomitant effects on leukocytes (e.g., inhibition of immune suppressive regulatory T cells).

Many chemokines are abundantly and concomitantly expressed in the TME and their function is regulated by complex mechanisms. In the latest years it has become clear that complexity is even higher because of the formation of heterocomplexes that exert antagonistic or synergistic effects on selected receptors.

D'Agostino et al. have collected the available scientific literature and their own experience on the phenomenon of heterocomplex formation, concentrating their investigation in cancer. The possible outcomes of heterocomplexes between chemokines, as well as between chemokines and inflammatory molecules (such as HMGB1) on the shaping of the TME is discussed.

Given their role in the pathomechanisms of tumor progression, chemoattractant receptors and their ligands constitute targets for the development of novel anti-tumor therapeutics. Two reviews provide comprehensive insight into the role of chemokines and receptors in tumor pathobiology and targeted treatments. Using publicly available data from The Human Protein Atlas, Vilgelm and Richmond constructed a heat map showing prognostic associations between 25 individual chemokines and 12 different types of cancer. Certain chemokines contribute to establishing a “T cell-inflamed” TME that is associated with improved prognosis, particularly when checkpoint inhibitor treatments are administered. The authors also describe a variety of chemokine-based countermeasures that can be deployed to populate an “immunologically cold” tumor with anti-tumor leukocytes. Poeta et al. focus on the role of chemokines and chemokine receptors in cancer with considerations on the possibility to be targets for cancer immunotherapy with emphasis on the possibility to optimize the anti-tumoral potential of the immune system. They present an overview on the current use of antagonists or inhibitors of chemokine receptors to treat different type of tumors both in preclinical model and clinical trial.

## Conclusions

It is our hope that this collection will serve to launch new studies that extend our understanding of chemoattractants in the pathomechanisms of tumor progression, and to inspire the discovery and development of new chemokine-focused treatments to make a real impact in the lives of cancer patients and their families.

## Author Contributions

GB and BZ have both contributed substantially to the work and approved it for publication.

### Conflict of Interest

The authors declare that the research was conducted in the absence of any commercial or financial relationships that could be construed as a potential conflict of interest.

